# ATP-Dependent Chromatin Remodeler CHD9 Controls the Proliferation of Embryonic Stem Cells in a Cell Culture Condition-Dependent Manner

**DOI:** 10.3390/biology9120428

**Published:** 2020-11-27

**Authors:** Hyunjin Yoo, Hyeonwoo La, Eun Joo Lee, Hee-Jin Choi, Jeongheon Oh, Nguyen Xuan Thang, Kwonho Hong

**Affiliations:** Department of Stem Cell and Regenerative Biotechnology and Humanized Pig Center (SRC), Konkuk University, Seoul 05029, Korea; Hyunjinyoo7@gmail.com (H.Y.); hyunwoo1001@naver.com (H.L.); ejlee1824@gmail.com (E.J.L.); heejinchoi1123@gmail.com (H.-J.C.); ocy0827@naver.com (J.O.); thang.nx1012@gmail.com (N.X.T.)

**Keywords:** CHD9, chromatin structure, ES cell, cell cycle, transcription

## Abstract

**Simple Summary:**

Chromodomain-helicase-DNA-binding protein 9 (CHD9) has been implicated in the regulation of gene expression, yet its precise role in the maintenance of mammalian embryonic stem cell (ESC) remains unclear. In the present study, we demonstrated that mouse CHD9 controls the cell cycle of ESCs in a cell culture condition-dependent manner by modulating the accessibility of transcription factors to their target genomic elements. Our study, therefore, has not only established how CHD9 finetunes chromatin structure during animal development but provided a potential target for genetic screening of aberrant development in in vitro produced embryos.

**Abstract:**

Emerging evidence suggests that chromodomain-helicase-DNA-binding (CHD) proteins are involved in stem cell maintenance and differentiation via the coordination of chromatin structure and gene expression. However, the molecular function of some CHD proteins in stem cell regulation is still poorly understood. Herein, we show that *Chd9* knockdown (KD) in mouse embryonic stem cells (ESCs) cultured in normal serum media, not in 2i-leukemia inhibitory factor (LIF) media, causes rapid cell proliferation. This is caused by transcriptional regulation related to the cell cycle and the response to growth factors. Our analysis showed that, unlike the serum cultured-*Chd9* KD ESCs, the 2i-LIF-cultured-*Chd9* KO ESCs displayed elevated levels of critical G1 phase regulators such as p21 and p27. Consistently, the DNA binding sites of CHD9 overlap with some transcription factor DNA motifs that are associated with genes regulating the cell cycle and growth pathways. These transcription factors include the cycle gene homology region (CHR), Arid5a, and LIN54. Collectively, our results provide new insights into CHD9-mediated gene transcription for controlling the cell cycle of ESCs.

## 1. Introduction

Chromodomain-helicase-DNA-binding (CHD) proteins, a subfamily member of the ATP-dependent chromatin-remodeling families, are largely evolutionary conserved. Eukaryotic CHDs have been implicated in diverse developmental processes including zygotic reprogramming, gastrulation and organogenesis [1,2,3]. CHD proteins (CHD1-9) are categorized into three subfamilies (subfamily I-III) based on their domain structures and sequence similarities, and their critical roles in stem cell function have been elucidated. Subfamily I proteins (CHD1 and CHD2) contain dual chromodomains at the N-terminal and an SNF2/helicase domain at the C-terminal region. CHD1 has been shown to be required for the maintenance of mouse embryonic stem cells (ESCs); it ensures the proper transcription of genes essential for pluripotency and prevents heterochromatin formation. Its loss leads to impaired ESC differentiation and somatic cell reprogramming into induced pluripotent stem (iPS) cells. CHD1 binding is highly correlated with markers of active transcription such as Polymerase II (Pol II) binding and H3K4me3 [4,5]. Mouse CHD2 physically interacts with organic cation transporter3/4 (OCT3/4) and prevents the formation of heterochromatin in ESCs by genes important for development. Its loss impairs late embryogenesis, neural circuit development, and long-term memory in mice [6,7].

The subfamily II proteins (CHD3-5) additionally contain a couple of PHD domains and form the nucleosome remodeling and deacetylase (NuRD) complex, a chromatin-remodeling complex that has histone deacetylase activity. CHD4 and 5 contribute to ESC differentiation into neural lineages through the transcriptional regulation of neuronal genes [8,9,10,11].

The subfamily III proteins (CHD6–9) are structurally similar to the subfamily II proteins, but they additionally contain SANT and BRK domains at the C-terminus [12]. Mutation experiments demonstrated that CHD7 regulates the expression of ESC-specific genes by promoting enhancer-promoter interactions along with other stem cell factors such as OCT3/4, SOX2, and NANOG [13]. CHD8 is necessary for the proliferation of cortical neural progenitors; it ensures the timely expression of cell cycle and neurogenic genes [14]. CHD9 (also known as the chromatin-related mesenchymal modulator) is found in the nucleolus, and its localization correlates with ribosomal transcription. It has been implicated in the differentiation of osteogenic cells: in this regard, it binds to skeletal tissue-specific promoters, including biglycan, core-binding factor subunit alpha 1 (CBFA1), collagen II, osteocalcin (OC), and myosin [15,16]. Indeed, a forced CHD9 overexpression in HEK293T cells induces transcriptional activation of RUNX2, a master regulator of osteoblast differentiation [17]. Recently, Alendar et al. showed that the function of mouse CHD9 is dispensable for development, and its loss in ESCs grown in ground-state conditions causes changes in gene expression [18]. However, a more detailed analysis is needed to elucidate the functions of CHD9 in the maintenance of pluripotency and the differentiation of ESCs.

The duration of the cell cycle and its turnover from one phase to the next in eukaryotes vary depending on the cell type. It has been shown that the cell cycle in mouse somatic cells takes approximately >16 h, whereas that in mouse ESCs takes ~8–10 h [19,20]. The cell cycle in mouse ESCs is unique and critical for the maintenance of pluripotency and differentiation [21]. One prominent feature of the cell cycle in ESCs is that the G1 phase is truncated [~2 h (in ESCs) vs. >10 h (in somatic cells)] and the S phase is slightly extended [~5 h (in ESCs) vs. ~7 h (in somatic cells)] [22,23]. It appears that the short G1 phase in ESCs is conserved in both human and monkey ESCs [24,25]. Furthermore, ground state ESCs maintained using two small-molecule inhibitors (PD0325901 and CHIR99021) and leukemia inhibitory factor (2i-LIF) have a much longer G1 phase than primed ESCs maintained in normal serum conditions [26,27]. In human ESCs, using the fluorescence ubiquitin cell cycle indicator (FUCCI) system, the G1 phase was also shown to be short; moreover, it increased rapidly upon differentiation. These studies suggest that the short G1 phase in ESCs is a necessary step for the maintenance of pluripotency by avoiding the expression of developmental genes and the acquisition of cell fate [25]. Nevertheless, little is known about the precise role of CHDs in controlling the cell cycle of mammalian ESCs with regard to the effects of serum vs. 2i-LIF.

Herein, we show that CHD9 silencing enhances ESC proliferation by changing the duration of the cell cycle phases. Our analysis also provides evidence that CHD9 modulates the status of chromatins in which transcription factors regulating ESC proliferation are accessible. Therefore, our study establishes new insights into CHD9-mediated gene transcription for controlling the cell cycle of ESCs.

## 2. Results

### 2.1. CHD9 is Dispensable for the Expression of Pluripotency Markers

**Figure 1:**
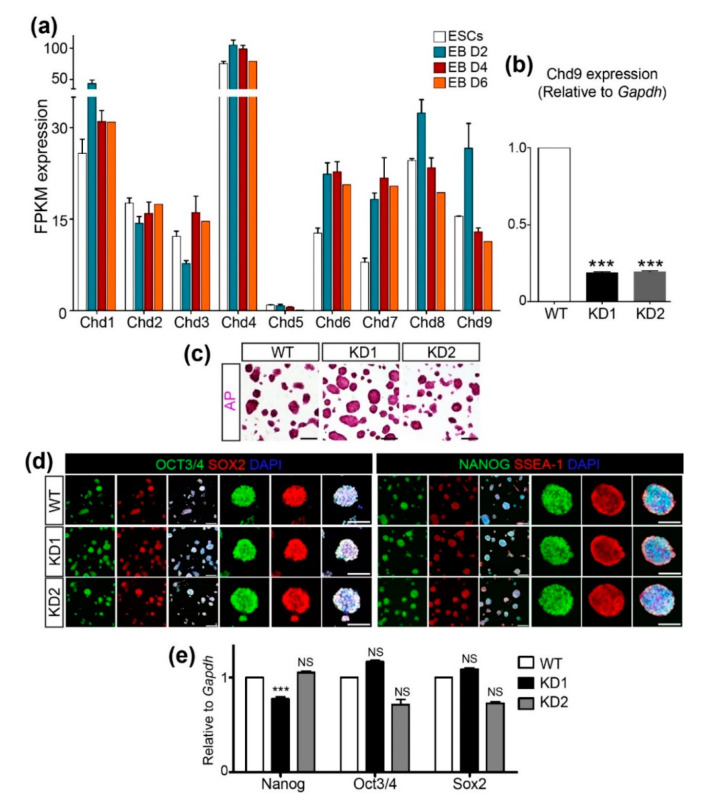
Chromodomain-helicase-DNA-binding (CHD)9 is dispensable for the pluripotency of embryonic stem cells (ESCs). (**a**) Expression values of the CHD family (*Chd1* to *Chd9*) in the ESCs and differentiated cells after 2, 4, and 6 d using RNA-Seq data (GSE114219). FPKM; Fragment per kilobase of transcript per million mapped reads. (**b**) shRNA-mediated *Chd9* KD efficiency in the ESCs was confirmed using qRT-PCR (n = 3). (**c**) ESCs were stained to screen for pluripotency through alkaline phosphatase activity. (**d,e**) The expression of the pluripotent markers (NANOG, OCT3/4, SOX2, and SSEA-1(IF)) was determined via immunofluorescence staining (**d**) and qRT-PCR (**e**), respectively. There was no significant difference in the transcription of *Nanog, Oct3/4*, and *Sox2* upon *Chd9* KD. The ESCs were immunostained with anti-NANOG, OCT3/4, SOX2, and SSEA-1 antibodies; scale bars in the merged images of low magnification in the left panels are 200 μm and of high magnification in the right panels are 100 μm.

To examine the expression pattern of *Chd* genes during mouse ESC differentiation, a publicly available RNA-Seq dataset (GSE114219) was obtained and re-analyzed to compare the fragments per kilobase of transcripts per million mapped reads (FPKM) values of *Chd1*~*9* (Figure 1a). *Chd4* expression was the most abundant, whereas *Chd5* expression was the least abundant during ESC differentiation. Among the subfamily III *Chd* genes, *Chd6* and *7* were induced immediately after differentiation and their expression levels were maintained at least up to D6 of differentiation. In contrast, a mild increment in *Chd8* and a robust increment in *Chd9* were detected at D2, though their levels decreased sharply at D4 and D6. Next, we sought to determine the role of CHD9 in the maintenance of ESC pluripotency using the short hairpin (sh) RNA KD approach. To this end, two shChd9 were designed, tested for their KD efficiency, and found to repress ~80% of endogenous *Chd9* expression (Figure 1b). Immunofluorescence, as well as AP staining, was performed to examine the levels of ESC markers, including OCT4, SOX2, NANOG, and SSEA-1. No gross changes in the levels of these markers were detected upon *Chd9* KD in the ESCs (Figure 1c,d). Moreover, the transcript levels of the pluripotent genes in the *Chd9* KD ESCs were indistinguishable from those in the control ESCs (Figure 1e). These data implied that CHD9 is dispensable for the expression of pluripotent markers.

### 2.2. Chd9 Silencing Promotes ESC Proliferation

**Figure 2:**
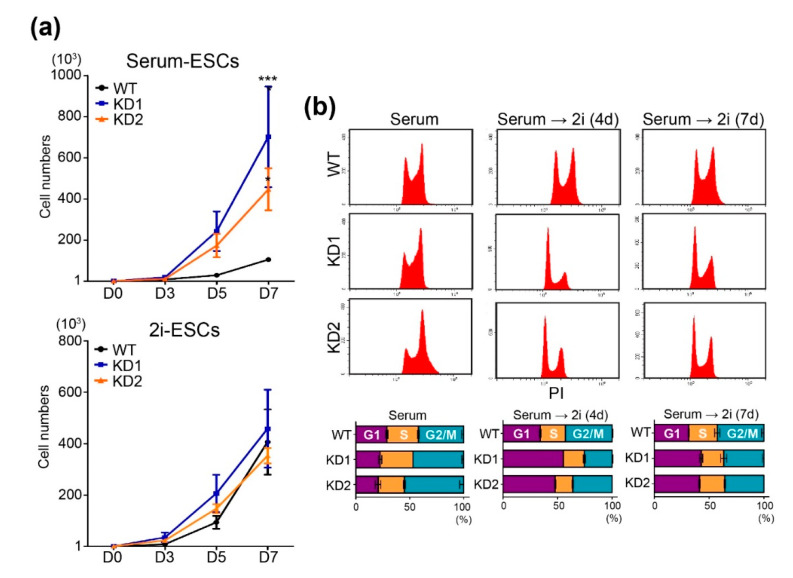
*Chd9* depletion changes cell cycle distributions in the serum-ESCs. (**a**) Cell numbers of the Wild type (WT) and KD ESCs were measured at 3, 5, and 7 d after seeding 1000 ESCs in either normal serum (serum-ESC) or 2i-LIF (2i-ESC) (n = 3). (**b**) Histogram graph and percentages of G1, S, and G2/M phases in the WT and KD ESCs analyzed by flow cytometry (n = 3).

Intriguingly, we found that the *Chd9*-depleted ESCs exhibited rapid cell proliferation. To precisely determine CHD9 function in ESC proliferation, cell growth was assessed up to 7 d after 1000 ESCs were seeded in normal serum media (“serum-ESCs”) or 2i-LIF media (“2i-ESCs”). As shown in Figure 2a, the *Chd9* KD serum-ESCs grew around two-times faster than the control serum-ESCs, whereas the *Chd9* KD 2i-ESCs grew at a normal rate. To confirm these findings, the *Chd9* KD ESCs were subjected to flow cytometric analysis. As shown in Figure 2b, the KD serum-ESCs exhibited a modestly shortened G1 phase compared to the control serum-ESCs, whereas the KD 2i-ESCs exhibited a significantly extended G1 phase compared to the control 2i-ESCs.

### 2.3. Chd9 Silencing Alters Gene Expression Related to the Cell Cycle

**Figure 3:**
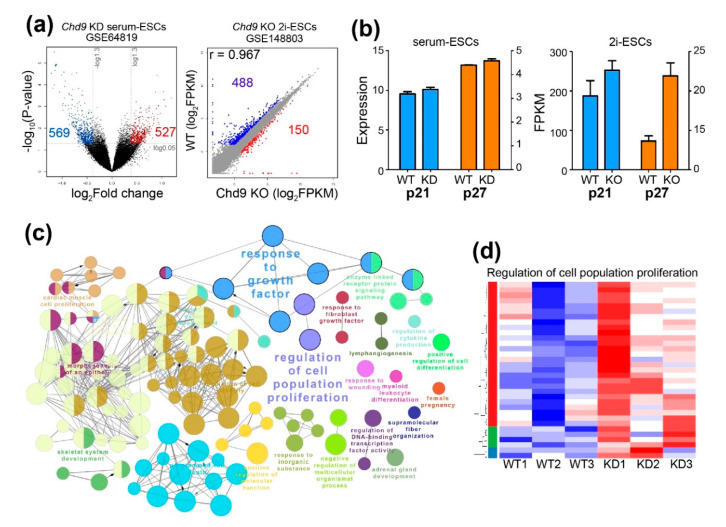
Chd9 depletion alters the expression of genes involved in the cell cycle in the serum-ESCs. (**a**) Volcano and scatter plot of microarray (GSE64819) and RNA-Seq (GSE148803) analyses. Upregulated genes and downregulated genes are indicated as red and blue dots, respectively. (**b**) qPCR analysis of p21 (encoded by *Cdkn1a*) and p27 (encoded by *Cdkn1b*) in the serum-ESCs and 2i-ESCs. (**c**) Gene ontology (GO) term analysis (biological processes (BP)) in the *Chd9* KD serum-ESCs. DEGs that were upregulated upon *Chd9* KD were used in the analysis. (**d**) Representative heatmap of the genes significantly altered by *Chd9* KD among the genes related to the “regulation of cell population proliferation” category.

To unravel the mechanism by which *Chd9* KD results in different patterns of cell proliferation depending on the culture conditions, publicly available data of a microarray (GSE64819) performed in *Chd9* KD serum-ESCs and RNA-Seq (GSE148803) performed in *Chd9* KO 2i-ESCs were re-analyzed. In the analysis of GSE64819, 569 downregulated DEGs and 527 upregulated DEGs were identified in the *Chd9* KD serum-ESCs. In contrast, 488 downregulated DEGs and 150 upregulated DEGs were identified in the *Chd9* KO 2i-ESCs (Figure 3a). Given that the upregulation of both p21 (encoded by *Cdkn1a*) and p27 (encoded by *Cdkn1b*) is required for the extension of the G1 phase in the 2i-ESCs, we examined the expression of these genes. As shown in Figure 3b, both genes were enhanced in the *Chd9* KO 2i-ESCs, whereas both genes were not changed in the *Chd9* KD serum-ESCs. A biological processes network analysis of upregulated DEGs showed that, among the various terms, genes related to “regulation of cell population proliferation” and “response to growth factor” were altered in the *Chd9* KD serum-ESCs (Figure 3c). However, these BPs were not identified in the *Chd9* KO 2i-ESCs. Although a batch effect was observed in the KD experiment, in general, most of the genes related to “regulation of cell population proliferation” were enhanced in the *Chd9* KD serum-ESCs (Figure 3d).

### 2.4. CHD9 Binding Regions Overlaps with the Transcription Factor Binding Sites Associated with the Regulation of the Cell Cycle

**Figure 4:**
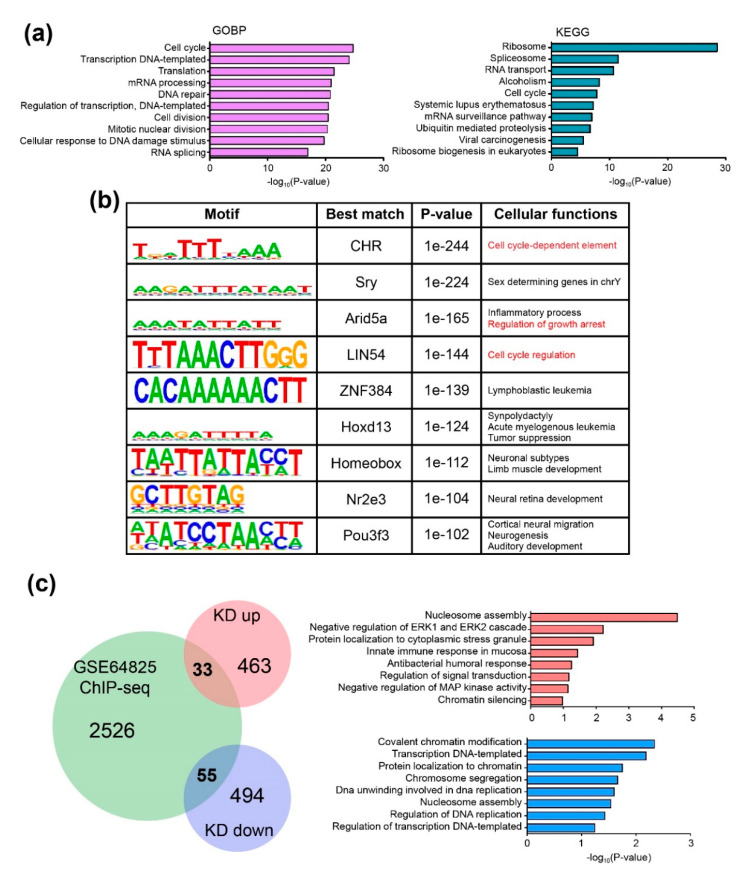
CHD9 binds directly to genes controlling the cell cycle. (**a**) Gene ontology (GO) term analysis for ChIP-seq data (GSE64825) for biological processes (BP) and KEGG. (**b**) De novo motif analysis of CHD9 ChIP-seq data showing the top nine significant motifs and best-matched transcription factors that bind to the motifs. Predicted cellular functions are shown. Cutoffs made by *p*-value < 1 × 10^−25^. (**c**) Venn diagram and GO term analysis for the identified common genes between ChIP-seq data and DEGs showing 33 genes commonly upregulated and 55 genes commonly downregulated. −log_10_(*p*-value) was used.

Next, to determine the CHD9 binding sites, publicly available data of a *Chd9* ChIP-Seq (GSE64825) carried out in mouse ESCs was re-analyzed. Our analysis identified 2614 genomic regions that *Chd9* directly binds to. Further downstream analyses, including gene ontology biological process (GOBP) and KEGG pathway, were performed. As shown in Figure 4a, the “cell cycle” (161 genes)-related GOBP was highly enriched. “Cell division” (109 genes) was also identified among the top 10 enriched GOBP in the analysis. Other GOBPs associated with transcription and translation were shown to be altered. The KEGG pathway analysis also identified gene expression- and cell cycle-related terms such as “ribosome” (71 genes) and “cell cycle” (38 genes). Next, we performed a DNA motif analysis to identify genomic regions that CHD9 binds to (Figure 4b). Then, the identified CHD9 binding motifs were examined to investigate whether transcription factor (TF) binding DNA sequences could be found in the motifs. The analysis showed that some CHD9 target sequences overlapped with known TFs that control the cell cycle. The predicted TFs included cell cycle gene homology region (CHR), AT-rich interaction domain 5A (Arid5a), and LIN54. In particular, the cell cycle-related CHR binding sequence, TTTAAA, was found in the *cyclin B1* (*Ccnb1*) promoter. CCNB1 is essential for the regulation of the cell cycle; it interacts with other proteins such as the dimerization partner (DP), RB-like, E2F4, and MuvB (DREAM) complex [28]. Next, we sought to identify direct CHD9 target genes by analyzing those that changed their expression level upon *Chd9* silencing. Thirty-three CHD9 target genes repressed by *Chd9* KD and 55 enhanced by *Chd9* KD were identified in the comparison between the microarray and ChIP-Seq data. Our GOBP analysis showed that genes associated with chromatin modification such as “nucleosome assembly,” “chromatin silencing,” and “regulation of transcription” were significantly affected (Figure 4c). These results suggest that the disruption of CHD9 results in changes in the chromatin structure at regions related to cell growth and causes aberrant proliferation.

## 3. Discussion

ATP-dependent chromatin remodelers have been shown to be implicated in the regulation of stem cell maintenance and differentiation, yet the biological function of CHD9 in ESCs is still unknown. In the present study, we demonstrated that CHD9 is required for the precise regulation of the cell cycle in mouse ESCs. Upon *Chd9* silencing, the serum-ESCs exhibited a mildly shorter G1 phase as well as a higher proliferation rate compared to the control ESCs. However, the extension of the G1 phase cannot be seen in the 2i-ESCs. The chromatin structure in ESCs is highly dynamic throughout the cell cycle. It is coordinated by stem cell factors such as OCT3/4 and SOX2 and closely related to the transcriptional regulation of genes that specify lineage commitment or maintain the self-renewal capacity. For example, the loss of OCT3/4 at the time of M-G1 transition leads to failure in ESC maintenance and an increased tendency of differentiation to neuroectoderm and mesendoderm [29]. This is most likely due to the increased chromatin accessibility near the differentiation genes in the absence of OCT3/4; it was observed even when OCT3/4 was degraded for a short time window during the M-to-G1 transition. This suggests that the coordinated regulation in the G1 phase by OCT3/4 and other TFs is critical for maintaining chromatin structure and pluripotency [30]. Consistent with published literature, *Chd9* KD altered the expression of genes involved in the cell cycle and responded to growth factors. In particular, our analysis showed that the expression of p21 and p27 was not altered in the *Chd9* KD serum-ESCs, whereas the levels of both p21 and p27 were enhanced in the *Chd9* KO 2i-ESCs. The data explain why only the *Chd9* KD serum-ESCs exhibited a truncated G1 phase and rapid proliferation. It has been shown that 2i-ESCs express high levels of p16, p21, and p27, and the depletion of either p21 or p27 independently leads to no change in the length of the G1 phase. The study demonstrated that both p21 and p27 are required for the extended G1 phase in the 2i-ESCs [26]. A recent report demonstrated that mouse CHD9 is dispensable for embryogenesis and postnatal development; however, it is required for gene expression [18]. Our analysis also showed that *Chd9* depletion changes gene expression without altering ESC maintenance and the expression of stem cell markers. Ooga et al. also showed that mouse CHD9 is necessary for growing oocytes to allow the loosening of the chromatin structure during the acquisition of pluripotent potential after fertilization [31]. Although our study has revealed a crucial role of Chd9 in the control of cell cycle depending upon ESC culture conditions, we cannot completely rule out the possibility that the identified Chd9 mechanism is due to the meta-analysis in the differentially produced cohorts (KD ESCs vs. KO ESCs) or different targeting sequences for Chd9 ablation. However, given that the direct comparison between KO and KD was only made in the determination of p21 and p27 levels, our bioinformatic analysis in Chd9 KD serum-ESCs is still worthwhile to explain mechanistic aspects of Chd9 functions. Furthermore, our study clearly demonstrates that Chd9 deficiency gives rise to a distinct role in the regulation of the ESC cell cycle at the cellular level.

Besides the regulatory TFs, the epigenetic landscape in the 2i-ESCs differs from that in the serum-ESCs. For example, the DNA methylation level is globally reduced in the 2i-ESCs compared to that in the serum-ESCs. Furthermore, histone H3K9me3 is enriched at the hypermethylated genomic regions, including endogenous retroviruses (ERVs) and imprinting genes [32]. Recently, Asenjo et al. demonstrated that the recruitment of Polycomb repressive complex 2 (PRC2) subunits to genes involved in the specification of lineages is tightly regulated across the cell cycle in mouse ESCs. EZH2, a catalytic subunit of the PRC2 complex, and JARID2, a protein enhancing PRC2 activity are enriched at bivalent gene promoters during the S and G2 phases, whereas EPOP, a PRC2 subunit activating bivalent genes, is enriched during the G1 phase [33]. Studies suggest that ATP-dependent chromatin remodelers play an important role in cell cycle control. Human INO80 was shown to negatively regulate p21 expression via p53-mediated binding to the p21 promoter [34]. *INO80* KD led to extended transition from G2/M to G1. The mammalian SWI/SNF complex (also known as the BAF complex) regulates the cell cycle depending on the composition of the complex subunits [35]. ARID1B (a subunit of the SWI/SNF complex)-depleted cells exhibited delayed DNA synthesis, whereas ARID1A (an alternative subunit of ARID1B in the SWI/SNF complex)-depleted cells led to a slight acceleration of the cell cycle [35]. The context-dependent subunit switch in the complex was shown to be important for the regulation of the expression of cell cycle regulators, including E2F genes [36]. Furthermore, BRG1 (a catalytic subunit of the SWI/SNF complex) regulates the proliferation of hepatocytes by modulating cell cycle-related genes such as *Ccnb1*, *Ccnb2*, *Cdk1*, and *Cdc20* [36]. Another context-dependent-Chd9 function in the regulation of the cell cycle was also demonstrated in a recent study. Xia et al., showed that CircPDZD8, a circular RNA, elevates *CHD9* expression by interfering with miR-197-5p in gastric cancer, and—interestingly—knocking down *CHD9* in gastric cancer cells decreases cell growth [37]. Additionally, some CHD proteins play roles in the regulation of the cell cycle and survival. CHD1 was shown to be essential for the maintenance of open chromatin and pluripotency in ESCs, and its silencing causes the aberrant regulation of pluripotency [5]. *Chd7* KD causes the inhibition of cell proliferation and neurogenesis in vitro by repressing *Hes5* and *N-myc* expression, whereas *Chd7* overexpression leads to the acceleration of cell proliferation in human ESC-derived neural stem/progenitor cells [38]. CHD7 is also critical for the proliferation of neural stem cells; its loss causes the activation of p53, resulting in human CHARGE syndrome [38,39]. *Chd8* mutation leads to induced apoptosis during early embryo development in mice by the activation of p53 [40]. CHD8 represses neuronal pathway-related transcripts, affecting brain development and synapse formation from induced pluripotent stem cells [41]. It is generally accepted that the synthesis of histone proteins is active during the S phase, such that nucleosome assembly quickly occurs in parallel with DNA replication [42]. Liu et al. also showed that the enrichment of histone H3K27 acetylation (H3K27ac) at proximal and distal enhancers ensures the maintenance of stem cell identity and the cell cycle during mitosis [43].

Our de novo motif analysis in CHD9 ChIP-Seq data showed that the CHD9 protein binds to genomic regions that overlap with cell cycle-related TFs binding DNA motifs, including CHR, LIN54, and Arid5a. It has been shown that CHR is present at the *Ccnb1* promoter and that p53-p21-DREAM functions through CHR to regulate the expression of some critical cell cycle regulators, including CCNA2, CCNB1, and POLD1 [44,45]. Moreover, CHR was shown to modulate the cell cycle or programmed cell death in humans and mice [46,47,48]. The DNA binding protein LIN54 also interacts with CHR elements in the cell cycle-associated promoter [48]. Arid5a regulates the quiescence stage by increasing the G0/G1 phase in human cancer cells; thus, it is regarded as a quiescence-associated marker [46]. Therefore, the ChIP-Seq analysis reveals how CHD9 deficiency leads to an enhancement of cell proliferation in the serum-ESCs.

## 4. Materials and Methods

### 4.1. ESC Culture

E14TG2a ESCs were obtained from ATCC (ATCC^®^ CRL-1821™, Manassas, VA, USA), and used for *Chd9* knockdown (KD) studies. ESCs were cultured in DMEM (Hyclone, Logan, UT, USA) supplemented with 15% fetal bovine serum (FBS) (Corning, NY, USA), L-glutamine (Gibco, Carlsbad, CA, USA), MEM non-essential amino acids (Gibco, Carlsbad, CA, USA), penicillin/streptomycin (Gibco, Carlsbad, CA, USA), β-mercaptoethanol (Gibco, Carlsbad, CA, USA), and LIF (Merck Millipore, Burlington, MA, USA) in a gelatin-coated dish. For the serum-free 2i culture conditions, the ESCs were maintained in DMEM/F12 (Gibco, Carlsbad, CA, USA): Neurobasal medium (Gibco, Carlsbad, CA, USA) (1:1) with N2 supplement (Gibco, Carlsbad, CA, USA), B27 supplement (Gibco, Carlsbad, CA, USA), penicillin/streptomycin (Gibco, Carlsbad, CA, USA), L-glutamine (Gibco, Carlsbad, CA, USA), 0.05% bovine serum albumin (BSA; Sigma, St Louis, MO, USA), 1 µm PD03259010 (Stemgent, Beltsville, MD, USA), 3 µm CHIR99021 (Stemgent, Beltsville, MD, USA), 150 µM monothioglycerol (Sigma, St Louis, MO, USA), and LIF (Merck Millipore, Burlington, MA, USA) in a gelatin-coated dish.

### 4.2. Chd9 KD in ESCs

To silence *Chd9* in the ESCs, lentiviral constructs for the short hairpin (sh) RNA of *Chd9* were purchased (RMM4534-EG109151, Dharmacon, Lafayette, CO, USA). The shRNA sequences used in the study are as follows; KD1 (5′–3′) AAAGCCTGATTTGTATGACCG, and KD2 (5′–3′) TAATCTGTAGACTTTAACTGC. The synthesized oligos were duplexed, sequenced, and transfected into 293T cells to produce lenti-shChd9 viral particles using the SuperFect Transfection reagent (Qiagen, Hilden, Germany). The supernatants harvested from the transfected 293T cells were pooled and concentrated using Amicon^®^ Ultra-15 Centrifugal Filter Unit, 100K (Merck Millipore, Burlington, MA, USA). Then, the ESCs were transduced using 10 μg/mL polybrene (Merck Millipore, Burlington, MA, USA) and selected by puromycin (2 μg/mL) treatment at 48 h after transduction for more than 7 d.

### 4.3. Alkaline Phosphatase (AP) Staining and Immunofluorescence

For immunofluorescence staining, the cells were fixed with 4% paraformaldehyde, blocked using blocking solution (3% BSA and 2% serum of host secondary antibodies with 0.1% Tween-20 in PBS), and incubated with primary antibodies overnight. Then, the cells were washed with 0.1% Tween-20 in PBS and incubated with fluorescent-conjugated secondary antibodies. After washing, the cell nuclei were counterstained with DAPI. The antibodies used in the study were; SSEA-1 (Merck Millipore, Burlington, MA, USA), SOX2 (Merck Millipore, Burlington, MA, USA), Oct-3/4 (Santa Cruz, Dallas, TX, USA), NANOG (Abcam, Cambridge, UK), donkey anti-rabbit or anti-goat IgG conjugated with Alexa Fluor 488, and anti-rabbit conjugated with Alexa Fluor 546 (Thermo Fisher Scientific, Carlsbad, CA, USA). For AP staining of the ESCs, the samples were fixed and stained using an Alkaline Phosphatase Staining Kit II (Stemgent, Beltsville, MD, USA) following the manufacturer’s instructions.

### 4.4. Proliferation, Flow Cytometry, and Quantitative Real-Time PCR Analyses

For the cell proliferation assays, 1000 cells were seeded in a cell culture dish and counted after 3, 5, and 7 d. For cell cycle analysis, the cells were fixed with 80% ethanol at 4 °C for more than 16 h and treated with RNase A solution (Sigma, St. Louis, MO, USA) for 2 h after washing with PBS. Then, the cells were stained with propidium iodide (PI) and analyzed flow cytometrically using CytoFLEX (Beckman Coulter, Brea, CA, USA). For quantitative real-time PCR (qPCR) analysis, total RNA was extracted from the cells (GeneAll, Seoul, South Korea) and used for the synthesis of cDNA (iNtRON, Seongnam, South Korea). qPCR was performed using SYBR green mix (KAPA, Wilmington, MA, USA) on a StepOnePlus™ System (Applied Biosystems, Waltham, MA, USA). The primers used in this study were: *Chd9* forward 5′-ACC CTT AAG GTT TCC ATC TG-3′, *Chd9* reverse 5′-TGA AGT TTC TGT ACC TGT TCC-3′; *Nanog* forward 5′- AAG CAG AAG ATG CGG ACT GT-3′, *Nanog* reverse 5′- ATC TGC TGG AGG CTG AGG TA-3′; *Oct3/4* forward 5′- CCA ATC AGC TTG GGC TAG AG-3′, *Oct3/4* reverse 5′- CCT GGG AAA GGT GTC CTG TA-3′; and *Sox2* forward 5′- GAA CGC CTT CAT GGT ATG GT-3′, *Sox2* reverse 5′- TTG CTG ATC TCC GAG TTG TG-3′.

### 4.5. RNA-Seq, Microarray, and ChIP-Seq Analyses

RNA-Seq data performed in mouse embryoid bodies (GSE114219) and *Chd9* knockout ESCs (GSE148803) were obtained from Gene Expression Omnibus (GEO). The quality of the sequencing reads of the data was controlled using FastQC [49], and the reads were aligned to UCSC mm10 genome assembly using the following STAR (v2.7.1a, NY, USA) [50] options: clip3pNbases argument set as 1. Cuffquant and Cuffnorm of the Cufflinks toolset (version 2.2.1, Seattle, WA, USA) [51] were used for the quantification of the normalized expression of each gene.

Microarray data performed in *Chd9* KD ESCs (GSE64819) were obtained from GEO. The microarray data were normalized and fitted into a linear model using the LIMMA (v3.40.6, QLD, Austria) tool [52] in R package (version 3.6.2, Vienna, Austria) [53]. Gene sets involved in the Gene ontology (GO) regulation of cell population proliferation and fold change of gene expression were obtained from Mouse Genome Informatics [54] and the LIMMA tool [55], respectively. The resultant heatmap showing the expression of the genes was generated using the heatmap2 function of the gplot (v3.0.4, Tel Aviv, Israel) [56] package and R tool. Differentially expressed genes (DEGs) were determined by comparison between the *Chd9* KD and control ESCs using LIMMA’s fold change and p-value data. With the list of DEGs, GO analysis was performed using the DAVID tool (version 6.8, Frederick, MD, USA) [57]. ClueGo (v2.5.1, Cordeliers Research Center, France) [58] plugged-in the Cytoscape (version 3.7.2, La Jolla, CA, USA) tool [59] was used to visualize the correlation of GO (biological pathway, BP) terms and KEGG pathways.

A publicly available CHD9 ChIP-Seq dataset (GSE64825) was downloaded from GEO. The read quality of ChIP-Seq was assessed using FastQC. Bowtie2 (v2.3.4.1, Maryland, USA) [60] was used to map the reads to UCSC mm9 mouse genome assembly using the following option: the trim3 1 option. The resultant files were used to call peaks in the MACS2 tool (v2.1.0, MA, USA) [61]. The peak regions calculated by MACS2 were used to find CHD9 binding motifs using the HOMER tool (v4.10.3, La Jolla, CA, USA) [62]. The MACS2 output bedgraph files were used as input for seqMINER (v1.3.4, CU de Strasbourg, France) [54] in which genomic regions surrounding all of the mm9 transcription start sites were visualized. Six gene clusters were calculated via the unsupervised clustering method. Genes in each cluster having signals around TSS were selected for comparison with DEGs from the microarray data. The commonly overlapping genes were used for GOBP analysis using DAVID.

### 4.6. Statistical Analysis

All the bar graph data were generated using Prism 5.0 (GraphPad Software, San Diego, CA, USA). Normality tests and equal variance tests were performed using SigmaPlot 12.0 (Systat Software Inc. Erkrath, German). If the data failed the normality test, a rank-sum test was used and plotted in a scatter plot. One-way ANOVA and two-way ANOVA with a Holm–Sidak test as post hoc analysis were used for the qRT-PCR data of the ectoderm marker and proliferation data, respectively. Unpaired t-test analysis was performed on the rest of the data. The error bars in the data represent the standard error of the mean (SEM). Statistical significance was determined at a *p*-value lower than 0.05(*) or 0.001(***).

## 5. Conclusions

In summary, our study demonstrates that CHD9 regulates ESC proliferation by regulating the expression of cell cycle-related genes in a cell culture condition-dependent manner. In addition, our analysis suggests that CHD9 directly modulates the accessibility of cell cycle-related TFs to their target genes in the serum-ESCs. The analysis of the exact mechanism and partner proteins of the CHD9 machinery, which regulates cell cycle-related genes, is warranted in follow-up studies.
